# Change of rapid temporal recalibration magnitude for audiovisual asynchrony with modulation of temporal binding window width: A preliminary investigation

**DOI:** 10.1177/20416695231193280

**Published:** 2023-08-18

**Authors:** Yasuhiro Takeshima

**Affiliations:** Department of Psychology, 12814 Hosei University, Japan

**Keywords:** multisensory/cross-modal processing, visuo-auditory interactions, temporal processing, synchrony, perceptual learning

## Abstract

The subjective synchrony perception for audiovisual stimuli is affected by previous temporal information. The point of subjective simultaneity is shifted toward the same asynchronous direction of audiovisual stimuli in a previous trial. This phenomenon is called “rapid temporal recalibration.” The factors that modulate the magnitude of rapid temporal recalibration have not been fully investigated. Previously, a positive correlation has been found between the magnitude of rapid temporal recalibration and the width of the temporal binding window (TBW). This preliminary study examined the causal relationship between TBW size and rapid recalibration magnitude using a single experimental group comparison design. In this experiment, the magnitude of rapid recalibration was compared before and after perceptual training, which narrowed the TBW width. The results indicated that the magnitude of rapid recalibration was reduced by perceptual training. Therefore, it was speculated that TBW size determined the magnitude of rapid recalibration. This causal relationship helps elucidate the mechanisms of the adaptation for temporal lags between visual and auditory sensations.

People perceive their external environment through sensory information. Temporal synchrony among different sensory inputs is a critical factor in the processing of multisensory integration. Many studies have demonstrated the necessity of simultaneous visual and auditory inputs for audiovisual integration. For example, in the backward masking paradigm, visual target detectability and discriminability are improved by simultaneous sounds (Bolognini et al., 2005; Chen & Spence, 2011). Other experimental tasks (e.g., attentional blink) are facilitated when a visual target is presented with simultaneous sounds (Olivers & Van der Burg, 2008).

Temporal synchrony perception for audiovisual stimuli is flexible. The subjective timing perceived as being maximally simultaneous (the point of simultaneity; PSS) often differs from the timing of physical synchrony (Vroomen & Keetels, 2010). The transmission times differ between light and sound, and the transduction times also differ between eyes and ears (King, 2005). Therefore, PSS is modulated by the distance to the source of the audiovisual stimuli (Engel & Dougherty, 1971; Sugita & Suzuki, 2003). In general, transmitting speed of light is faster than that of sound; thus, observers tend to perceive it as simultaneous when visual stimuli are presented prior to auditory stimuli. In addition, the size and intensity of visual stimuli also modulated the PSS score (Kanai et al., 2017; Krueger Fister et al., 2016). Moreover, observers judge it as simultaneous when there is a certain range of lag between visual and auditory stimuli. This tolerance range is known as the temporal binding window (TBW). The brain is considered to treat the two information streams as belonging to the same event within the range of TBW. For example, audiovisual stimuli are perceived simultaneously with sounds of 100–200 ms that precede and follow them (Dixon & Spitz, 1980; Guski & Troje, 2003). The TBW size differs between the visual field in which the visual stimuli are presented (Takeshima, 2021). This tolerance range is also associated with the processing of audiovisual interactions. The illusions induced by audiovisual integration occur even when there are presentation lags between visual and auditory stimuli (i.e., stimulus onset asynchronies; SOAs). These lags (SOAs) approximately consist of the tolerance range of subjective synchrony (e.g., Remijn et al., 2004; Takeshima & Gyoba, 2013).

Temporal recalibration is a function of adapting to temporal lags between visual and auditory stimuli. Repeated exposure to asynchronous audiovisual stimuli shifts PSS in the same direction as the leading stimuli of exposure (Fujisaki et al., 2004; Vroomen et al., 2004). This phenomenon is known as temporal recalibration. Van der Burg et al. (2013) indicated that temporal recalibration occurs without repeated exposure periods in the simultaneous judgment (SJ) task, which they termed “rapid temporal recalibration.” Temporal recalibration with repeated exposure shows a large but decaying recalibration effect after exposure; although a rapid temporal recalibration produces a large transient effect that follows previous asynchrony (Van der Burg et al., 2015).

Rapid temporal recalibration is a fast-acting adaptation to asynchronous inputs of audiovisual stimuli with unique characteristics. First, rapid temporal recalibration depends only on the physical temporal information (i.e., SOAs) of the preceding audiovisual stimuli. For example, rapid temporal recalibration occurs regardless of inconsistent visual (i.e., color or orientation) or auditory (i.e., frequency) features (Harvey et al., 2014), actors (individuals who appear in the brief movie clip; Van der Burg & Goodbourn, 2015), or spatial locations (stimulus is presented in a different location: Ju et al., 2019) between the previous and current trials. Second, the PSS is shifted based on the physical timing, rather than the perceived timing of the preceding audiovisual stimuli in rapid temporal recalibration (Van der Burg et al., 2013, 2018). Simon et al. (2017) reported that intertrial PSS shifts are reflected in late-evoked components of event-related potentials. Therefore, rapid temporal recalibration could be induced by the change in the synchrony perception criterion attributed to low-level temporal information of audiovisual events (Takeshima, 2020).

Although the factors involved in the occurrence of rapid temporal recalibration have been clarified, the factors that modulate recalibration magnitude have not yet been investigated. Van der Burg et al. (2013) reported a positive correlation between the magnitude of rapid recalibration and TBW size. If there is a causal relationship between these two variables, then narrowing TBW size should decrease the magnitude of rapid recalibration. Thus, this study investigates whether manipulation of TBW size changes the magnitude of rapid recalibration. Perceptual training for SJ judgment of audiovisual stimuli narrows TBW size (Powers et al., 2009). Changes in TBW size by perceptual training are not due to response bias (Powers et al., 2009), and the cortices associated with this training effect have been clarified (Powers et al., 2012).

In summary, this study compared the magnitude of rapid recalibration before and after perceptual training for SJ judgment of audiovisual stimuli in a single experimental group; thus, conducting as a preliminary investigation. The aim of this study was to examine the factors that define the magnitude of rapid recalibration for audiovisual stimuli. This preliminary investigation helps elucidate the mechanisms of adapting to temporal lags between visual and auditory stimuli.

## Method

### Participants

Twenty-six individuals (14 men and 12 women; mean age = 21.62 ± 2.13 years) participated in this experiment. The sample size was determined by conducting an a priori power analysis using G*Power Version 3.1 (Faul et al., 2009) to detect the main effect of time with an effect size (*f*) = 0.30, significance level (*α*) = .05, and power (1-*β*) = .80 (with a minimum sample size of 23 participants). Participants were recruited to meet the required sample size. All participants orally reported normal or corrected-to-normal vision and normal hearing. Participants provided written informed consent before participation. This study was approved by the ethics committee of the Department of Psychology, Doshisha University (no. 201906).

### Apparatus

Stimuli were generated and controlled using a custom-made program written in MATLAB (The MathWorks, Inc.), Psychtoolbox (Brainard, 1997; Kleiner et al., 2007; Pelli, 1997), and a laptop personal computer (MacBook Pro, Apple). Visual stimuli were displayed on a 21-inch CRT display (Trinitron CPD-G520, Sony; resolution: 1024 × 768 pixels; refresh rate: 100 Hz). Auditory stimuli were conveyed through an audio interface (Clarett 2Pre, Focusrite) and headphones (MDR-CD900ST, Sony). The simultaneity of the visual and auditory stimuli was confirmed using a digital oscilloscope (DS-5424A, Iwatsu). The experiment was conducted in a dimly lit room with 39.8 dB (A) background noise. Participants viewed the monitor binocularly at a distance of 70 cm with their heads stabilized on a chin rest.

### Stimuli

White Gaussian probes were used as visual stimuli. The size of the visual stimulus was approximately 2.0° (*σ* = 0.3°) in diameter, with a duration of 50 ms. The fixation point was a white cross (159.51 cd/m^2^) with a diameter of 1.1° in diameter. These stimuli were presented on a gray background (29.37 cd/m^2^). The auditory stimulus was a pure tone of 500 Hz with a duration of 50 ms (including ramp times of 5 ms at the beginning and end of the sound wave envelope), and the sound pressure level was set at 55 dB (A). There were nine SOAs between the visual and auditory stimuli: ±510, ±260, ±130, ±50, and 0 ms (negative SOAs indicate that the auditory stimulus was presented before the visual stimulus and vice versa) in the pre- and post-sessions. In the feedback training session, seven SOAs were observed between visual and auditory stimuli: ±150, ±100, ±50, and 0 ms.

### Pre- and post-SJ session

**Figure 1.**
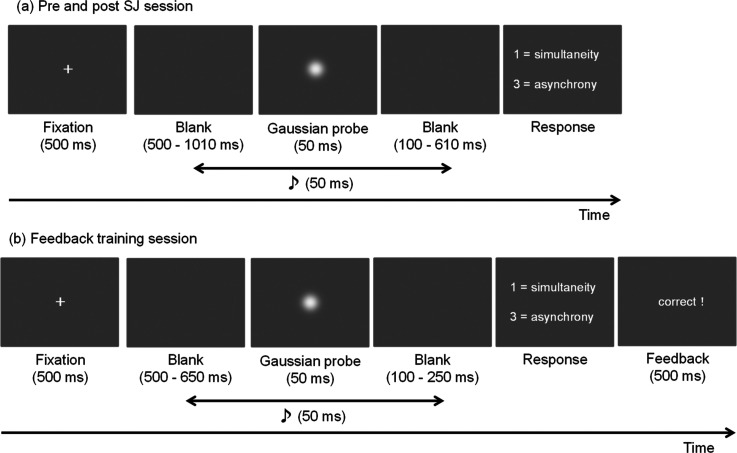
(a) Schematic representation of the pre- and post-simultaneity judgment sessions’ trial sequence. (b) Schematic representation of the feedback training session's trial sequence.

A schematic representation of the trial is shown in Figure 1a. The trials were initiated by pressing “0” on the keyboard. Each trial consisted of a 500 ms fixation followed by blank and visual stimulus presentations. The duration of the blank displays was a fixed length of 500 ms, plus an additional SOA when the auditory stimulus was presented prior to the visual stimulus (i.e., 500 ms at the shortest and 1010 ms at the longest). During the target display period, a visual stimulus was presented for 50 ms accompanied by an auditory stimulus randomly selected from a range of nine SOAs. After presenting the visual stimulus, participants were instructed to judge whether the presentation of the visual and auditory stimuli was simultaneous by pressing “1” for simultaneity and “3” for asynchrony. Before the pre-SJ session, participants performed 20 practice trials. The pre- and post-SJ sessions consisted of five blocks each. One block consisted of 100 trials (0 ms SOA was 20 trials repetitions + other SOA conditions 10 trials repetitions). Each participant completed 500 trials in one session. Participants took short breaks between blocks.

### Feedback training session

A feedback training session was conducted between pre- and post-SJ sessions. A schematic representation of the trial is shown in Figure 1b. The trial sequence was similar to the pre- and post-SJ sessions, except that the feedback display was presented for 500 ms. During the target display period, a visual stimulus was presented for 50 ms with an auditory stimulus randomly selected from the range of seven SOAs. During the feedback display period, participants were presented with either the phrase “correct” or “incorrect” corresponding to the correctness of their response: however, a Japanese word (“*seikai*” or “*fuseikai*”) with the same meaning was displayed in practice. The feedback training session consisted of three blocks, each. One block consisted of 120 trials (0 ms SOA was 60 trials repetitions + other SOA conditions were 10 trials repetitions). Each participant completed 360 trials during this session.

## Results

The proportion of “simultaneous” responses was calculated for each condition in the pre- and post-SJ sessions. An intertrial analysis was conducted to examine whether the modality order in a given previous trial (*t*-1) affected the distribution of simultaneity responses in the current trial (*t*). Furthermore, the distribution of perceived simultaneity as a function of SOA was compiled for each participant and session separately, given that in some cases trial t-1 exhibited either a negative (i.e., audition leads) or positive (i.e., vision leads) SOA. The synchrony distributions were then fitted with the following Gaussian function to each participant's data based on minimization of the root-mean-square-error (RMSE) to compute the PSS:

(1) $$P\;(\mathrm{response}|\mathrm{SOA})\;=\mathrm{Amplitude}\cdot e^{\left[-.5{\left(\frac{\mathrm{SOA}\;-\mathrm{PSS}}{\mathrm{Sigma}}\right)}^{2}\right]}$$

**Figure 2.**
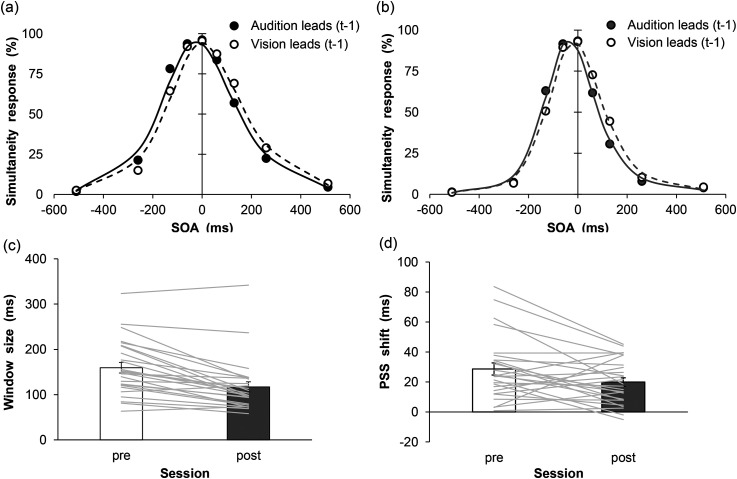
Results of temporal binding window size and rapid recalibration magnitude in pre- and post-simultaneity judgment sessions. Mean percentage of simultaneous responses in the (a) pre- and (b) post-simultaneous judgment (SJ) sessions. (c) Mean estimated window size. (d) Mean estimated rapid recalibration magnitude. Gray lines indicate the individual data. Error bars represent standard errors of the mean (*n* = 26).

The SOA parameter was equal to that specified under the experimental conditions (from −510 to +510 ms). The parameters of amplitude, PSS, and sigma values were estimated, and the amplitude and sigma values were restricted to values greater than 0. The results of the analysis are shown in Figure 2a and 2b, which demonstrate the mean percentages of simultaneity responses as a function of session, modality order, and SOA with fitted psychometric functions (pre/audition leads: mean RMSE = 0.07 ± 0.03, pre/vision leads: mean RMSE = 0.08 ± 0.03, post/audition leads: mean RMSE = 0.07 ± 0.02, post/vision leads: mean RMSE = 0.07 ± 0.03).

The TBW size (mean of the sigma values for both audition and vision leads on trial *t*-1) was computed in each session (Figure 2c). The results showed that the TBW size was smaller in the post-SJ than in the pre-SJ session (*t* (25) = 5.89, *p* < .001, *g* = 0.70). Additionally, the magnitude of the recalibration effect (PSS when vision leads − PSS when audition leads on trial *t*-1) was calculated in each session (Figure 2d). The result indicated that the rapid recalibration effect was lower in the post-SJ condition than in the pre-SJ condition (*t* (25) = 2.40, *p* = .02, *g* = 0.47).

**Figure 3.**
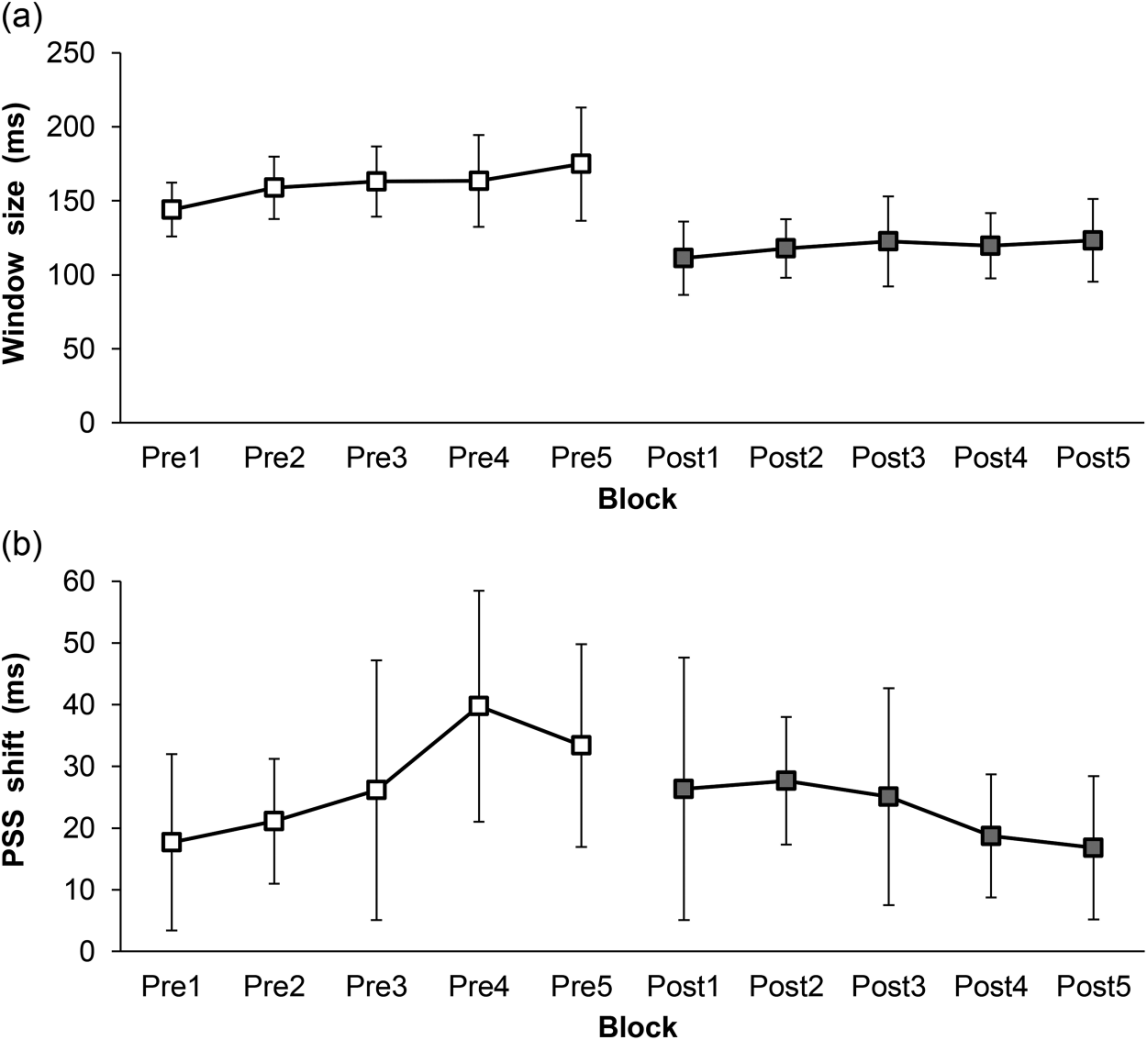
Results of temporal binding window (TBW) size and rapid recalibration magnitude in each block. (a) Mean estimated window size. (b) Mean estimated rapid recalibration magnitude. Error bars represent a 95% confidence interval (*n* = 26).

Moreover, to exploratory investigate the time course of changes of TBW size and the recalibration effect, these values were computed for each block in the pre- and post-SJ sessions. The results are shown in Figures 3a and 3b. Since this analysis is an exploratory analysis, the results of visual inspection of mean data are reported. The TBW size tended to slightly increase with block in the pre-SJ session. In the post-SJ session, the change in the TBW size with block was negligible. The TBW in block 1 of the post-SJ session was smaller than in block 5 of the pre-SJ session. Contrastingly, the recalibration effect largely increased between blocks 3 and 4 in the pre-SJ session. In the post-SJ session, the recalibration effect decreased since block 2. The recalibration effect was lower in block 1 of the post-SJ session than in block 5 of the pre-SJ session.

## Discussion

This preliminary study examined the causal relationship between TBW size and the magnitude of rapid recalibration using perceptual training in an audiovisual SJ task. As in a previous study (Powers et al., 2009), the TBW width narrowed after perceptual training that provided correct feedback for the response of the SJ task. The magnitude of PSS shifts owing to rapid temporal recalibration decreased after perceptual training. Moreover, both TBW size and the magnitude of rapid recalibration changed between pre- and post-sessions (i.e., before and after perceptual training). There is a positive correlation between TBW size and the magnitude of rapid recalibration (Van der Burg et al., 2013). Therefore, these results suggest that narrowing the TBW width induces a decreasing in the magnitude of rapid temporal recalibration.

A reasonable interpretation for a causal relationship would be that the change in TBW size by the perceptual training determined the change in the magnitude of rapid recalibration, rather than *vice versa*. There might be an opposite causal relationship between TBW size and the magnitude of rapid recalibration: reducing the magnitude of rapid recalibration owing to perceptual training decreased TBW size. However, rapid temporal recalibration is induced by physical timing information of audiovisual stimuli (Van der Burg et al., 2013, 2018). The current perceptual training provided participants with only the correct judgment information for the SJ task. Thus, the temporal information of physical timing would not be altered by perceptual training. This study interprets that perceptual training decreased TBW size rather than the magnitude of rapid recalibration.

Unfortunately, this study could not conclude that there is a causal relationship between TBW size and the magnitude of rapid recalibration. Because there is no control group in this experiment, it could be also interpreted that the TBW size and magnitude of rapid recalibration was changed by repeatedly performing an SJ task (i.e., a practice effect). However, decreasing the magnitude of rapid recalibration by repeating an SJ task has not yet been reported. Van der Burg et al. (2015) showed the change in the magnitude of rapid recalibration within the session, but not between sessions. If a repeated SJ task decreases the magnitude of rapid recalibration, this finding would inform future experiments of rapid temporal recalibration. To clarify the factor of the change in the magnitude of rapid recalibration, future studies should conduct present feedback training experiment including both experimental and control groups.

If the TBW size can determine the magnitude of rapid recalibration, then the range of the PSS shift owing to rapid temporal recalibration is suggested to be within the width of the TBW. This means that there is a limitation that the magnitude of PSS shift owing to rapid recalibration is constrained within the width of the TBW. This limitation would be associated with individual differences in the magnitude of rapid temporal recalibration. For example, the magnitude of rapid recalibration is changed throughout the life span (Noel et al., 2016). Noel et al. (2016) showed that both TBW size and the magnitude of rapid recalibration for flash-beep stimuli exhibit a U-shape pattern as a function of age. The causal relationship between TBW size and the magnitude of rapid recalibration could explain this change throughout the life span. Therefore, this study helps elucidate the mechanisms of the adaptation for temporal lags between light and sound.

This study speculates about other individual differences in the magnitude of rapid temporal recalibration. Turi et al. (2016) reported that rapid temporal recalibration cannot be observed in individuals with autism spectrum disorder (ASD). However, Weiland et al. (2022) showed that the rapid temporal recalibration occurred in both groups with and without ASD. Both Turi et al. (2016) and Weiland et al. (2022) found no difference in the TBW size between the groups with and without ASD. If the TBW size determines the magnitude of rapid recalibration, the difference in this magnitude should not be observed between these two groups. Weiland et al. (2022) indicated that the differences in these results may be attributed to differences in the age groups of the sample; the age group of Turi et al. (2016) is younger than that of Weiland et al. (2022). Thus, the present study speculates that the magnitude of rapid recalibration is independent of TBW size for younger individuals with ASD but becomes more dependent on it with age.

A limitation of this study is that there was only one experimental group (i.e., performing perceptual training) in the current experimental design. As noted above, all participants repeatedly performed the SJ task; therefore, it is possible that repetition of the SJ task, rather than perceptual training, reduced the width of TBW (i.e., a practice effect). An additional experiment should be conducted with an experimental design that includes both experimental and control groups. Second, this study only examined the decreasing direction of the magnitude of rapid recalibration. The perceptual training proposed by Powers et al. (2009) can only reduce the TBW size. Additionally, perceptual training that broadens the TBW size has not yet been devised. Future studies should examine whether the magnitude of rapid recalibration increases owing to the broadening of the TBW size by devising new perceptual training methods. Third, the mathematical relationship between the change in TBW size and that in the magnitude of rapid recalibration is unresolved. There is a linear relationship (i.e., a positive correlation) between individual differences in TBW size and the magnitude of rapid recalibration (Van der Burg et al., 2013). However, the relationship between the amount of change in TBW size and the magnitude of rapid recalibration after perceptual training has not been quantified. Presenting a mathematical model that quantitatively represents the magnitude of rapid recalibration should also be conducted in future studies.
